# Mixed-Mode Solar Drying and its Effect on Physicochemical and Colorimetric Properties of Zompantle (*Erythrina Americana*)

**DOI:** 10.1007/s11130-024-01147-0

**Published:** 2024-02-08

**Authors:** Octavio García-Valladares, Alfredo Domínguez-Niño, Ana María Lucho-Gómez, Andrea Gail Jiménez-Montiel, Arcel Siareth Rodríguez-Mendoza, Beatriz Castillo-Téllez, Mario Luna-Flores, Margarita Castillo-Téllez

**Affiliations:** 1 Departamento de Sistemas Energéticos, Instituto de Energías Renovables-UNAM, Temixco, Morelos Mexico; 2 Consejo Nacional de Humanidades, Ciencia y Tecnología-Cátedra CONAHCYT, Dirección Adjunta de Desarrollo Científico, Mexico City, Mexico; 3 Departamento de Agua y Energía, Centro Universitario de Tonalá, Universidad de Guadalajara, Tonalá, Jalisco Mexico; 4 Departamento de Ingeniería en Procesos Bioalimentarios, Universidad Tecnológica del Centro de Veracruz, Cuitláhuac, Veracruz, Mexico; 5 Facultad de Ingeniería, San Francisco de Campeche, Universidad Autónoma de Campeche, Campeche, Mexico

**Keywords:** Edible flower, Zompantle, Solar drying, Ultraviolet radiation, Proteins, Solar energy

## Abstract

A mixed-mode solar drying was developed to evaluate the physicochemical and colorimetric properties of Zompantle (*Erythrina americana*). A 2^2^-factorial design was used; the operation mode (mesh shade and direct) and airflow (natural convection and forced convection) were established as factors in this design. The initial moisture content in the Zompantle flower was reduced from 89.03% (w.b) to values that ranged from 3.84% to 5.84%; depending on the operation mode of the dryer, the final water activity ranged from 0.25 to 0.33. The Zompantle’s components as proteins (4.28%), antioxidant activity (18.8%), carbohydrates (4.83%), fat (0.92%), fiber (3.71%), ash (0.94%), and total soluble solids (3°Brix) increased as the water was evaporated during the drying. The increment in the Zompantle’s components depends on the operation mode; in direct mode and natural convection, the proteins, antioxidant activity, carbohydrates, fat, fiber, ash, and total soluble solids were 6.99%, 61.69%, 79.05%, 1.20%, 3.84%, 8.70%, and 45 °Brix, respectively. The total drying efficiency was 14.84% with the direct mode and natural convection (DM-NC) and 17.10% with the mesh shade and natural convection (MS-NC). The Hue angle measures the property of the color; the indirect mode and natural convection keep the hue angle close to the initial value (29.2 °). The initial chroma value of the Zompantle flower was 55.07; the indirect mode and natural convection kept high saturation (37.58); these dry conditions ensured a red color in the dehydrated Zompantle. Dehydrated Zompantle’s flowers could have several practical applications, such as an additive in traditional Mexican cuisine.

**Supplementary Information:**

The online version contains supplementary material available at 10.1007/s11130-024-01147-0.

## Introduction

Zompantle, *Erythrina americana* Mill, is an endemic tree; it was a sacred tree for Aztecs, who took advantage of its medical properties that are still used nowadays [1]. There are 113 species of Erythrina worldwide, mainly in tropical and subtropical regions, and it is commercialized in South America, Central America, and West Africa. Twenty-seven of these species are found in regions of Mexico such as México City, Guanajuato, Hidalgo, Jalisco, Michoacán, Morelos, Oaxaca, Chiapas, Guerrero, Nuevo León, Puebla, Querétaro, Tabasco, Tamaulipas, Veracruz and Yucatán [2]. *Erythrina americana* Mill, also known as Zompantle, colorin, cochizquilitl, gasparito, pemuche, machete, pichoco, and alcaparras, among other nicknames, belongs to the leguminous family [3]. The Zompantle’s blossoms are fried or boiled in stews or sauces and are appreciated because of their high protein and lipid content, representing a great food alternative. However, due to its high content of humidity, Zompantle is highly transitory and has a propensity to decomposition reactions; regularly, the studied species of edible flowers have short commercial longevity, which varies from 4 to 10 days, while the maximum total longevity varies from 6 to 14 days [4]. Consumption of dry flowers has increased due to their nutritional and medicinal value; therefore, drying has been the most used among numerous preservation methods. In the open literature, some studies have been reported on the drying of edible flowers such as pumpkin flower [6], walnut male flower [7], daylilies [8], magnolia liliiflora [9], marigold flower, jasmine, carnation [10]. However, conventional methods such as freeze drying, combined far-infrared radiation with hot air convection, hot air drying, microwave drying, vacuum drying, hybrid drying, and dehumidified drying have been reported [11]. Nowadays, the application of solar technologies in food preservation has been developed. Solar drying is an effective, safe, and low-priced food preservation technique with minimal environmental impact. This process is more economical than storage because dried flowers occupy less space, weigh less, and do not require refrigeration [5]. A solar dryer uses solar radiation, forced convection, and natural ventilation to decrease the humidity content in a product [12]. This technology can be used to obtain quickly processed products, store them for long periods, and use them conveniently to manufacture formulated foods. Drying decreases the water-related activities of plants and consequently inhibits the growth of microorganisms while decreasing the rate of the biochemical reactions, thus extending the shelf life of the products at room temperature [13]. Solar dryers can be classified into two kinds: active and passive. Fans are integrated into the cabin in active systems to advance the humidity drag. In contrast, air flows naturally in passive systems because of lift forces due to density differences due to a temperature gradient. In direct solar dryers, part of the radiation that the product is exposed to is absorbed by the product itself, which increases the temperature and the product’s water evaporation. The humidity exchange with the material’s interior to the immediate environment depends on the diffusion phenomena. At the same time, mass diffusivity relies on different factors such as shape, structural components, and humidity content. On the other hand, in indirect dryers, the air that passes through the collector is heated and transported to the drying chamber to transfer the thermal energy to the dehydrated product. For this research, an active dryer mixed type was used; during the drying process, the solar irradiance was attenuated by using a mesh shade not only to decrease the drying temperature but also to evaluate the effect of solar and ultraviolet irradiance on physicochemical properties of Zompantle during the drying process. To the best of our knowledge, there are no reports in the literature about solar drying of the Zompantle flower. Zompantle’s flowers could have several practical applications, for instance, as an additive not only in traditional Mexican cuisine but also for dishes such as pasta, creams, flours, and even formulated foods. The impact and degree of incidence of this research is that results that were obtained in this study will be applied to specific problems such as the use of fossil fuels with rising prices, which deteriorate the environment, food waste in rural areas of the country, hunger and energy poverty; development of community centers for solar dehydration of food in rural areas to reduce food waste, seeking to contribute to reducing environmental impact, increasing regional community economic development, as well as greater production and availability of nutritious products in the market and at the rural families. Therefore, this work aimed to evaluate the effect of mixed-mode solar drying on the physicochemical and colorimetric properties of Zompantle the flower.

## Material and Methods

Detailed description is provided in the supplementary information file.

## Results and Discussion

### Characterization of Zompantle Flower

The initial moisture content and water activity of the zompantle flower were 89.03% (w.b.) and 0.970, respectively (Table 1); zompantle is highly transitory and has a propensity to decomposition reactions; regularly, the studied species of edible flowers have short commercial longevity, which varies from 4 to 10 days, while the maximum total longevity varies from 6 to 14 days [4].

**Table 1:** Physicochemical analysis of zompantle

Analysis	Mean values^±SD^	Analysis	Mean values^±SD^
Moisture content (w.b)	89.03^±2.20^	Fiber (%)	3.71^±0.1746^
Water activity	0.970^±0.07^	*L*	34.36^±0.79^
Total soluble solids (°Brix)	3.0^±0.01^	a	45.93^±4.42^
Total Proteins (%)	4.28^±0.13^	b	30.35^±5.88^
Ash (%)	0.94^±0.04^	Chroma	55.07^±0.28^
Antioxidant activity (%)	18.8^±0.02^	Carbohydrates (%)	4.83^±0.04^
Fat (%)	0.92^±0.04^	Hue	33.32^±1.38^

±SD (Standard deviation) *L* (lightness), *a* (greenness-Redness), *b* (Yellowness-Blueness)

According to Hamrouni [13], flowers have a high moisture content of up to 80% (w.b). Isis [14], Pinedo et al [15], and Lara et al [16] reported 87.02, 88.1, and 86.6% (w.b) of moisture content in the zompantle; however, the moisture content of Zompantle ranges from 85.25 to 91.77 depending on the post-harvest phase. The colorimetric analysis in the zompantle flower showed positive values in *a* (45.93) and *b* (30.35) parameters (Table 1); according to the Hunter system, the redness and yellowness are represented by *a* and *b* values on the positive side; therefore, a dominant red color in zompantle flower was observed. On the other hand, the color property is measured by the Hue angle; in this case, the zompantle flower showed a hue angle of 33.32°; according to the literature, when the hue angle ranges from 0° to 90 °, the color pass from red to yellow color in the product [17] in this case, the hue is in the red zone. The chroma value in the zompantle flower was 55.07, indicating that the red color is pure and intense. The literature reports that pigment is related to purity or color saturation (chroma), which increases with increasing pigment concentration [6]. Pinedo et al [15] said a chroma value of 15.85%, a hue angle of 45.31, and a lightness of 54.55; these results infer that the zompantle flower was less red because of the high lightness and low chroma. The proximal analysis in the raw zompantle flower showed 4.287% of proteins, 0.9455 ash, 0.9237% of fat, 3.0 °Brix of total soluble solids, the antioxidant activity of 18.8%, carbohydrates of 4.83%, and 3.71% of fiber; the results were close in some properties to the proximal analysis of zompantle reported by Sotelo et al [18] (4.10 °Brix, 87.60% moisture, 8.97% of ash content, 13.69 of fiber, and 56.64 of carbohydrates). Lara et al[16] reported the proximal composition of zompantle (86.6 g/100 g of moisture content, 26.2 g/100 g of proteins, 2.8 g/100 g of fat, 12.7 g/100 g of fiber, 5.8 g/100 g of ash, and 62.1 g/100 g of nitrogen-free extract). Some properties of the zompantle flower were reported by Pinedo et al [15] and López et al [19].

## Drying Kinetics

The solar drying process of Zompantle flower was carried out on 31 January, 1st – 2nd of February, 7th–8th of February, and 9th–10th of February, 2023, using an active dryer mixed type (Supplementary information).

**Fig. 1:**
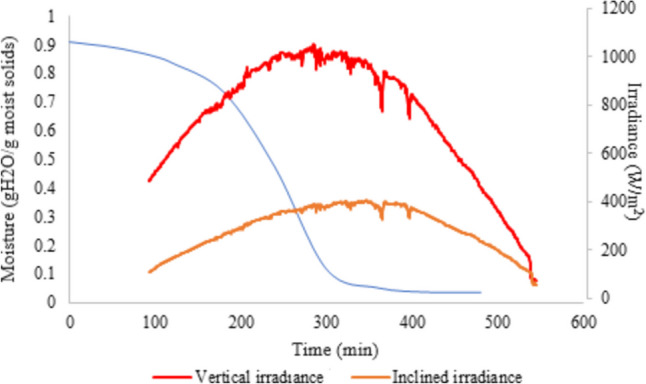
Drying kinetics of Zompantle (*Erythrina americana*) carried out in a mixed-type solar dryer on the day 31 January 2023

The test began from 9:00 a.m. to 18:00 p.m.; two days were necessary to dry the Zompantle flower, except for the experimental test on 31 January (Fig. 1). Figure 1 shows the drying kinetics of Zompantle; the Zompantle was dehydrated in 480 min (8 h) the day 31 January, 660 min (11 h) the day 7th–8th of February, 660 min (11 h) the day first – 2nd of February, and 720 min (12 h) the day 9th–10th of February (Supplementary information).

**Fig. 2:**
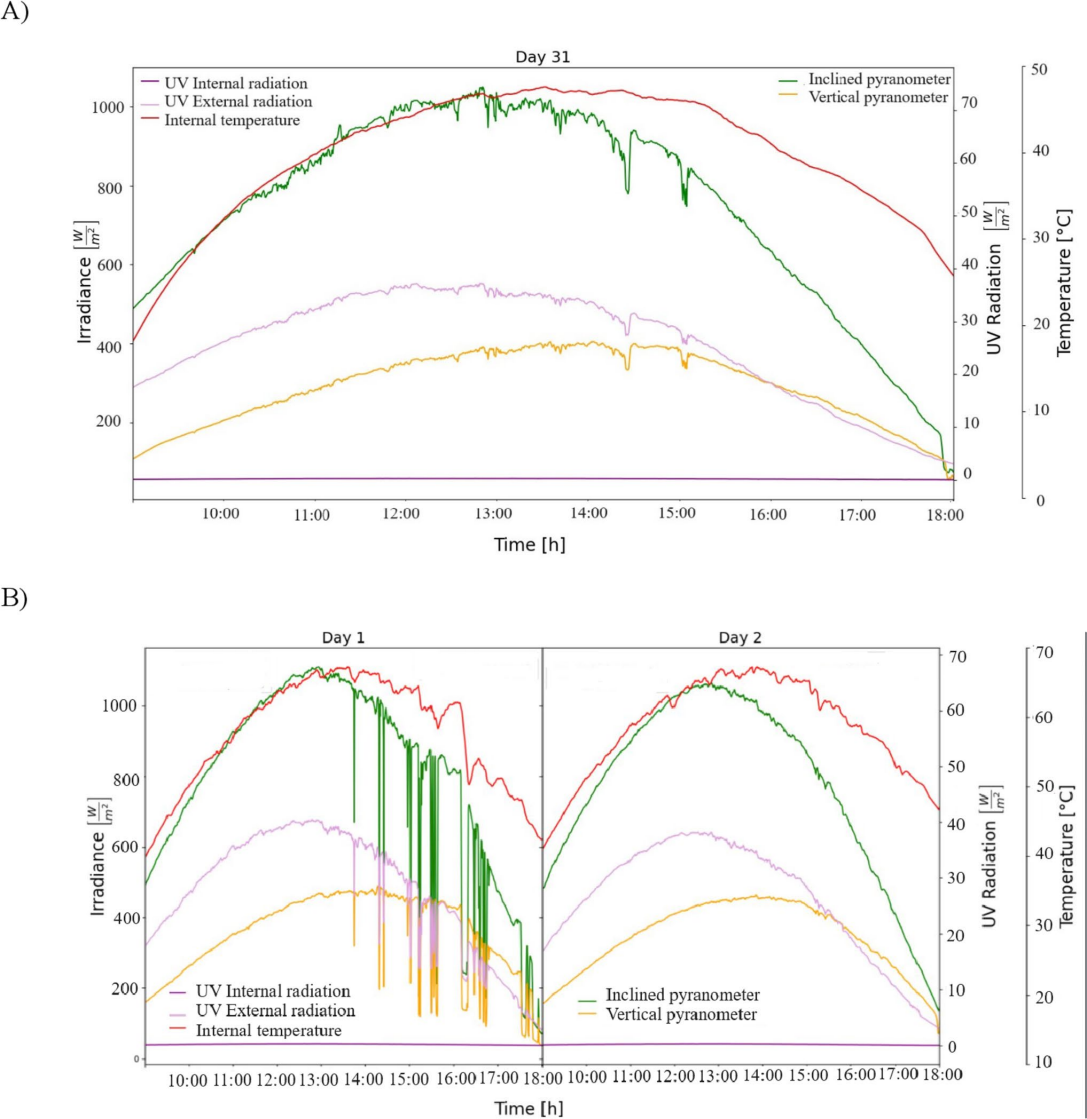
Temperature, solar, and ultraviolet radiation during the drying process of zompantle flower carried out the days: **A** 31 January, **B** 1st – 2nd of February

The difference in the drying time was affected by the mesh shade and the forced convection. The maximum temperatures registered inside the dryer were 74.36 °C in direct mode and natural convection (31st of January) (Fig. 2A) and 67.73 °C with forced convection (1st – 2nd of February) (Fig. 2B). As seen from the Figures, when the dryer operates in direct mode, the maximum irradiance is 1050 W/m^2^ and 1112.01 W/m^2^, respectively.

**Fig. 3:**
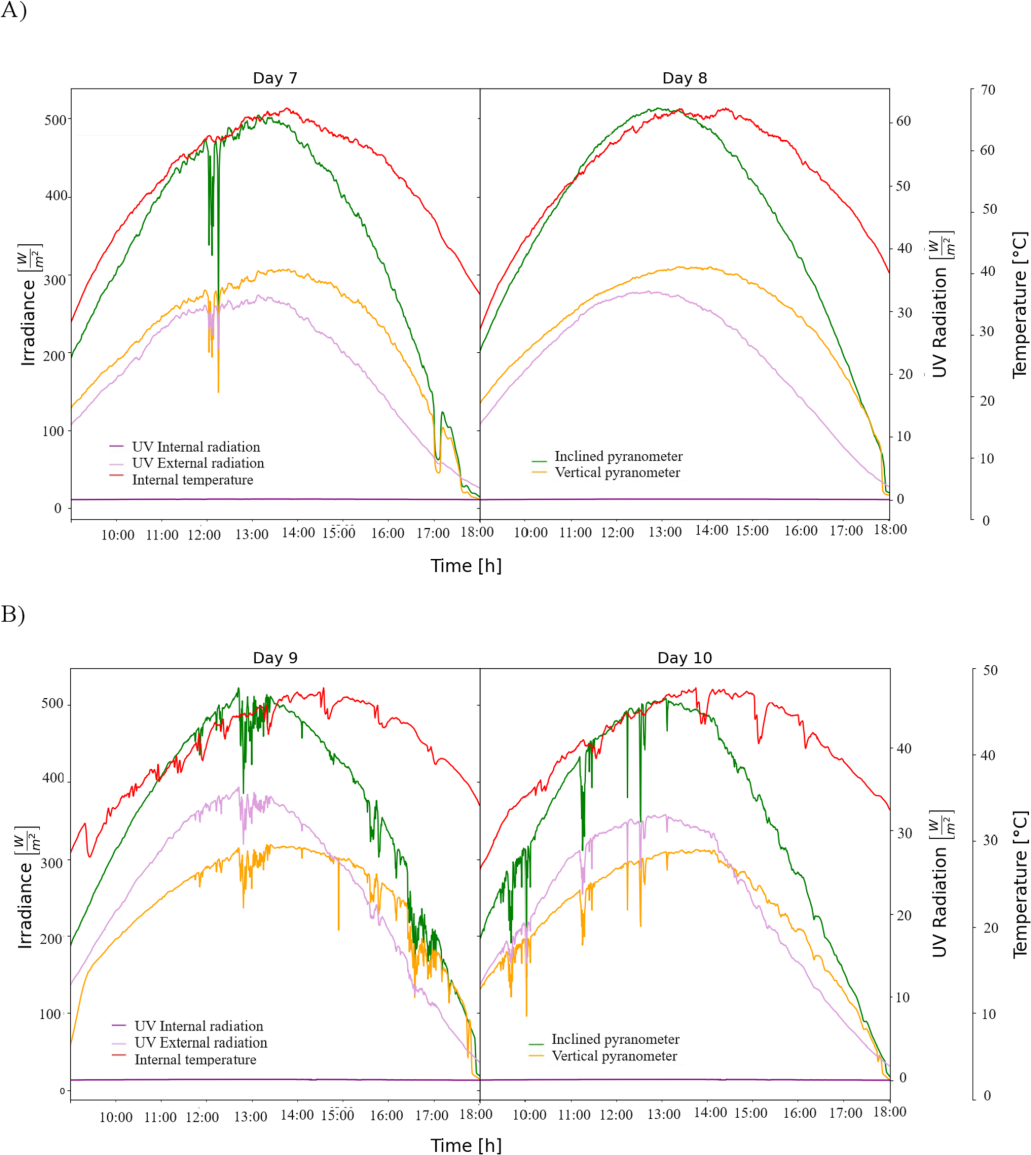
Temperature, solar, and ultraviolet radiation during the drying process of zompantle flower carried out the days: **A** 7th–8th of February, and **B**) 9th–10th of February

The solar dryer receives the sun’s radiation in the horizontal and vertical plane, favoring homogeneous drying by the temperature increment. When the dryer operates by using a mesh shade and natural convection, the solar irradiance decreased to 504.22 and 490.29 W/m^2^, and the maximum drying temperature was 62.28 °C (7th–8th of February) (Fig. 3A) and 44 °C with forced convection (9th–10th of February) (Fig. 3B).

In all drying conditions, the maximum external ultraviolet radiation ranged from 31.99 to 40.40 W/m^2^ depending on the ambient conditions; however, although the cover of the drying chamber was made of polycarbonate with ultraviolet protection, the ultraviolet radiation detected inside the dryer ranged from 0.1236 to 0.2873 W/m^2^ depending the operation mode of the dryer. Rodríguez et al [20] reported that the cover material of solar dryers influences fruit properties due to the ultraviolet radiation and temperature; their results demonstrated that dried samples of strawberries in polyethylene cover favored the remaining 40.7% of anthocyanins, whereas in cellular polycarbonate where the ultraviolet radiation was zero only 15.5% of total anthocyanins were retained. In this study, the optical transmittance of cellular polycarbonate is too low; therefore, the ultraviolet radiation inside the dryer was near zero; therefore, the effect of ultraviolet radiation was not significant because the polycarbonate material limited the passage of ultraviolet radiation inside the dryer.

In the literature, some studies have been reported about the solar drying of edible flowers; García et al [6] said the drying kinetics of pumpkin flowers by using modified solar dryers; in their results, eight to fifteen hours were needed to dry the pumpkin flower depending to the drying cover employed. Fernandes [21] reported three days of sun drying in *Robinia pseudoacacia* at 35 °C. The drying time, as well as the retention of the nutritional components of the edible flower, depends on the drying technology and ambient conditions used to dry the sample [22].

### Moisture Content and Water Activity of Dehydrated Zompantle

The initial moisture content in the zompantle flower was reduced from 89.03% to values that ranged from 3.84 to 5.84%, depending on the operation mode of the dryer (Supplementary Table 1) (Table 2).

**Table 2:** Physicochemical analysis of dehydrated Zompantle

Analysis	Mean values^±standard deviation^			
	MS-NC	DM-NC	MS-FC	DM-FC
Drying time (h)	11	8	12	11
Moisture content (%)	3.84^±1.38^	4.06^±0.43^	5.38^±0.14^	5.84^±0.46^
Water activity	0.27^±0.01^	0.26^±0.02^	0.25^±0.01^	0.33^±0.05^
Lightness	36.08^±3.77^	23.08^±1.91^	27.89^±1.49^	26.91^±0.01^
Chroma	37.58^±1.84^	9.87^±1.60^	14.77^±0.72^	16.01^±0.01^
Hue	31.65^±6.68^	40.94^±6.17^	52.46^±5.53^	45.08^±0.06^
Color difference ∆E	29.2^±0.76^	39.69^±2.77^	37.56^±3.75^	40.63^±2.25^
Total proteins (%)	7.65^±0.11^	6.99^±0.08^	5.06^±0.08^	4.94^±0.13^
Fat (%)	2.30^±0.13^	1.20^±0.14^	1.42^±0.11^	1.62^±0.15^
Fiber (%)	4.93 ± ^0.14^	3.84 ± ^1.38^	5.89 ± ^0.10^	5.56 ± ^0.10^
Ash (%)	8.08^±0.09^	8.70^±0.12^	8.41^±0.05^	8.59^±0.09^
Antioxidant activity (%)	35.68^±0.02^	61.69^±0.02^	51.14^±0.02^	42.44^±0.02^
Carbohydrates (%)	78.13^±0.02^	79.05^±0.02^	79.73^±0.02^	79.01^±0.02^
Total soluble solids (°Brix)	36^±0.01^	45^±0.01^	33^±0.01^	39^±0.01^

*MS* Mesh shade, *NC* Natural convection, *DM* Direct mode, *FC* Forced convection

As seen from Table 2, when the dryer was operated with the mesh shade and direct mode with forced convection, high values in moisture content were obtained (5.38 and 5.84%). On the other hand, low moisture values (3.84 and 4.06%) were observed with natural convection using a mesh shade to attenuate the solar irradiance and direct operation mode. This behavior relates to high temperatures reached inside the dryer with natural convection. The analysis of variance (Supplementary Table 2) showed that the factors did not affect the zompantle’s final moisture content. Usually, food with high moisture content is very prone to spoilage; in this case, the dried zompantle flower can be considered safe for storage because the final moisture was reduced by less than 10% [23]. The initial water activity of the pumpkin flower was 0.970, and it was controlled by reducing the moisture content. As seen from Table 2, the final water activity ranged from 0.25 to 0.33; this water activity means that chemical reactions and biological processes will not take place. The analysis of variance revealed that drying conditions did not affect the final water activity in the dried product.

### Colorimetric Analysis in Dehydrated Zompantle Flower

The zompantle flower showed an initial lightness value of 34.36 (Table 1); this colorimetric property tends to decrease to 23.08 and 27.89 (Table 2) when the operation mode was DM-NC and MS-FC. The analysis of variance showed that only the operation mode significantly affected the lightness. The lightness decreases due to the product’s water content loss because the moisture content affects the reflectance color; therefore, the zompantle tends to be dark. On the other hand, the lightness increased to 36.08 in MS-NC. Changes in food color result from many factors, including the modification of cellular structure, changes in pH, degradation of carotenoids, and loss of water content. Ferouali [24] reported a decrease in color parameters (*L*, *a*, and *b*) by using indirect solar drying at 40, 50, and 60 °C in *Punica granatum* flower; the best preservation of color was at 40 °C. During the heating, the samples can develop degradation of pigments; firstly, a slightly yellow color is observed; then, the sample turns red, and if the sample contains high total soluble solids, a brown color can be observed [25]. The Hue angle measures the property of the color, and it is expressed in degrees.

The initial hue angle was 33.32°; this value means that the zompantle flower is near to red color. The analysis of variance revealed that the interaction effect of operation mode and airflow significantly affected the Hue color. The Hue angle starts at the +a axis and is expressed in degrees; 0° is red, and an increase in Hue angle means that the color goes to red, orange, and yellow (90°, +b). According to Table 2, the MS-NC keeps the hue angle close to the initial value (29.2 °). Chroma is an intensity measurement, taking values from 0 to 60. The initial chroma value of the zompantle flower was 55.07. According to the results, the sample becomes dark when the chroma values decrease; conversely, the color will be purer and more intense with high chroma. In this case, the MS-NC keeps high saturation (37.58) with a high hue angle (31.65). These dry conditions ensure a red color in the dehydrated zompantle. The variation between the raw and dried samples is known as the total color difference (∆E). As seen from Table 2, the ∆E values ranged from 29.2 to 40.63. According to the descriptive levels in ∆E, a range up to 12 means a noticeable difference concerning the standard [26]. Although the lowest color difference (29.2) was observed in MS-NC, the color change resulted appreciably in this case.

### Physicochemical Properties in Dehydrated Zompantle

The initial protein content in the raw zompantle flower was 4.29% (Table 1); however, all treatments observed an increment in this property at the end of the drying process. Table 2 shows that protein content ranged from 4.94% to 7.65% in the dehydrated zompantle; the highest protein content (7.65%) was observed in MS-NC. The analysis of variance showed that the independent variables affected significantly this response variable. The drying conditions conserved the protein content better than MS-NC. The initial fat content in the zompantle flower was 0.9237%, and this component ranged from 1.20 to 2.30%, depending on the drying conditions. In general, flowers apport low-fat content, and as a result, they are considered low-calorie foods. Pinedo [15] reported the physicochemical properties in edible flowers of wild plants of Mexico as *A. salmiana, A vera, E. Americana, and M. geometrizans*; in their investigations, 1.58, 2.95, 1.05 g, and 1.69 g/100 g of ether extract, respectively were reported. Ahluwalía [27] used different drying methods (vacuum and cabinet dried) in the dehydration of Marigold petals (*Tagetes erecta*); their results demonstrated that some constituents, such as proteins, ash, and fiber, increased, whereas properties such as antioxidant activity and total phenolic content decreased significantly. Ahluwalía [27] reported an increment in protein content from 2.0 to 4.28% in vacuum drying and 3.40 in cabinet drying; on the other hand, the ash content increased from 0.45 to 3.20% in vacuum drying and 2.02 in cabinet drying; finally, the fiber content increased from 1.67 to 10.9 and 12.50% in vacuum and cabinet drying, respectively. Some researchers have reported that the food’s components increase as the water evaporates during the drying [6, 28]. Ferouali [24] reported that the best preservation of bioactive molecules in *Punica granatum* can be obtained at 40 °C; however, Fernandes [21] mentioned that high carotene content in marigold can be obtained at 60 °C. Table 2 shows an increment in fiber, ash content, antioxidant activity, carbohydrates, and total soluble solids observed in the components of zompantle. The fiber in the raw zompantle flower was 3.71, and this property ranged from 3.84 to 5.89% in the dehydrated flower; the antioxidant activity increased from 18.8% to values ranging between 35.68 to 61.69%, and the total soluble solids increased from 3.0% to 33–45%, depending on the drying conditions. The analysis of the results showed that the MS-NC and DM-NC preserved the components of zompantle better.

The supplementary information file provides mathematical modeling of drying kinetics and energy efficiency.

## Conclusions

In this study, the drying conditions that conserved better the physicochemical properties of the Zompantle flower were by using the mesh shade to attenuate the solar irradiance and natural convection. At these conditions, the total efficiency was 17.10%, the maximum drying temperature was 62.28 °C, the complete proteins were 7.65%, fat 2.30%, fiber 4.93%, ash 8.08%, and total soluble solids 36 ° Brix. However, the antioxidant activity can be increased using direct mode and natural convection (61.69%) and mesh shade but with forced convection (51.14%). Using the dryer with the mesh shade and polycarbonate with ultraviolet protection can attenuate the amount of ultraviolet radiation inside the dryer (0.1249 W/m^2^–0.1131 W/m^2^) and decrease the drying temperature. The moisture content in the zompantle flower was reduced from 89.03% to values that ranged from 3.84% to 5.84%. The final water activity ranged from 0.25 to 0.33; this water activity means that chemical reactions and biological processes will not take place. An increment in total soluble solids, protein content, fat, ash, and fiber better preserved the zompantle components. With the mesh shade and natural convection, keep a high saturation (37.58) with a high hue angle (31.65); these dry conditions ensure a red color in the dehydrated zompantle. This study suggests using the solar dryer in indirect mode; in this operation mode, the Zompantle flower is not exposed to direct radiation; it is only dehydrated with the air that passes through the collector to the drying chamber. Dehydrated Zompantle’s flowers could have several practical applications, for instance, as an additive not only in traditional Mexican cuisine but also for dishes such as pasta, creams, flours, and even formulated foods.

### Supplementary Information

ESM 1 (DOCX 1197 kb)

## Data Availability

Data supporting the findings of this study are available from the corresponding author upon reasonable request.
