# Supplementary material for: Mixed-Mode Solar Drying and its Effect on Physicochemical and Colorimetric Properties of Zompantle (Erythrina Americana)
Source: Plant Foods Hum Nutr. 2024 Feb 8;79(1):194–201. doi: 10.1007/s11130-024-01147-0 (PMC10891242; doi:10.1007/s11130-024-01147-0)
Supplement: Supplementary file 1 — (DOCX 1197 kb) [file 11130_2024_1147_MOESM1_ESM.docx]

**SUPPLEMENTARY INFORMATION**

**Mixed-mode solar drying and its effect on physicochemical and colorimetric properties of Zompantle (*****Erythrina Americana*)**

Octavio García-Valladares^a^, Alfredo Domínguez-Niño ^a,b*^, Ana María Lucho-Gómez^a^, Andrea Gail Jiménez-Montiel^a^, Arcel Siareth Rodríguez-Mendoza^a^, Beatriz Castillo-Téllez^c^, Mario Luna-Flores^d^, Margarita Castillo-Téllez^e^

^a^Instituto de Energías Renovables-UNAM, Departamento de Sistemas Energéticos, Temixco, Morelos, México. ^b^Consejo Nacional de Ciencia y Tecnología-Cátedra CONAHCYT, Dirección Adjunta de Desarrollo Científico, Mexico City, Mexico. ^c^Centro Universitario de Tonalá, Universidad de Guadalajara, Departamento de Agua y Energía, Tonalá, Jalisco, México. ^d^Universidad Tecnológica del Centro de Veracruz, Departamento de Ingeniería en Procesos Bioalimentarios, Cuitláhuac, Veracruz, México. ^e^Universidad Autónoma de Campeche, Facultad de Ingeniería, San Francisco de Campeche, Campeche, Mexico.

*Corresponding author: aldoni@ier.unam.mx (A. Domínguez-Niño)

Octavio García Valladares <http://orcid.org/0000-0001-9478-4157>

Alfredo Domínguez Niño http://orcid.org/0000-0001-5411-8264

Ana María Lucho Gómez http://orcid.org/0000-0001-9775-4113

Andrea Gail Jiménez Montiel http://orcid.org/0000-0001-5411-8264

Arcel Siareth Rodríguez Mendoza http://orcid.org/0009-0004-1346-9518

Beatriz Castillo Téllez http://orcid.org/0000-0003-3747-6320

Mario Luna Flores <https://orcid.org/0000-0003-4272-4560>

Margarita Castillo Téllez https://orcid.org/0000-0001-9639-1736

***2.1 Raw material***

The Zompantle flower was obtained from a local producers' market located in Ixtaczoquitlán, Veracruz, México (18.85 Latitude, 97.0617 Longitude 18 ° 51', 8'' N at 1186 mamsl). The zompantle was cleaned, washed, and disinfected before drying.

***2.2 Dryer description***

For this research, an active dryer mixed type was used; it has an integrated fan to favor the humidity drag and has combined equipment; the dehydrator can directly absorb the heat, and the process is improved by adding an air heater flat plate solar collector. The drying chamber contains ten trays (0.63 x 0.43 m) in five levels; each tray has 0.0025 x 0.007 m perforations. The cover of the drying chamber was made of polycarbonate of 6 mm with ultraviolet protection, and the dryer was operated in direct mode (Figure 1A) and with mesh shade to attenuate the solar irradiance and to decrease the drying temperature (Figure 1B).


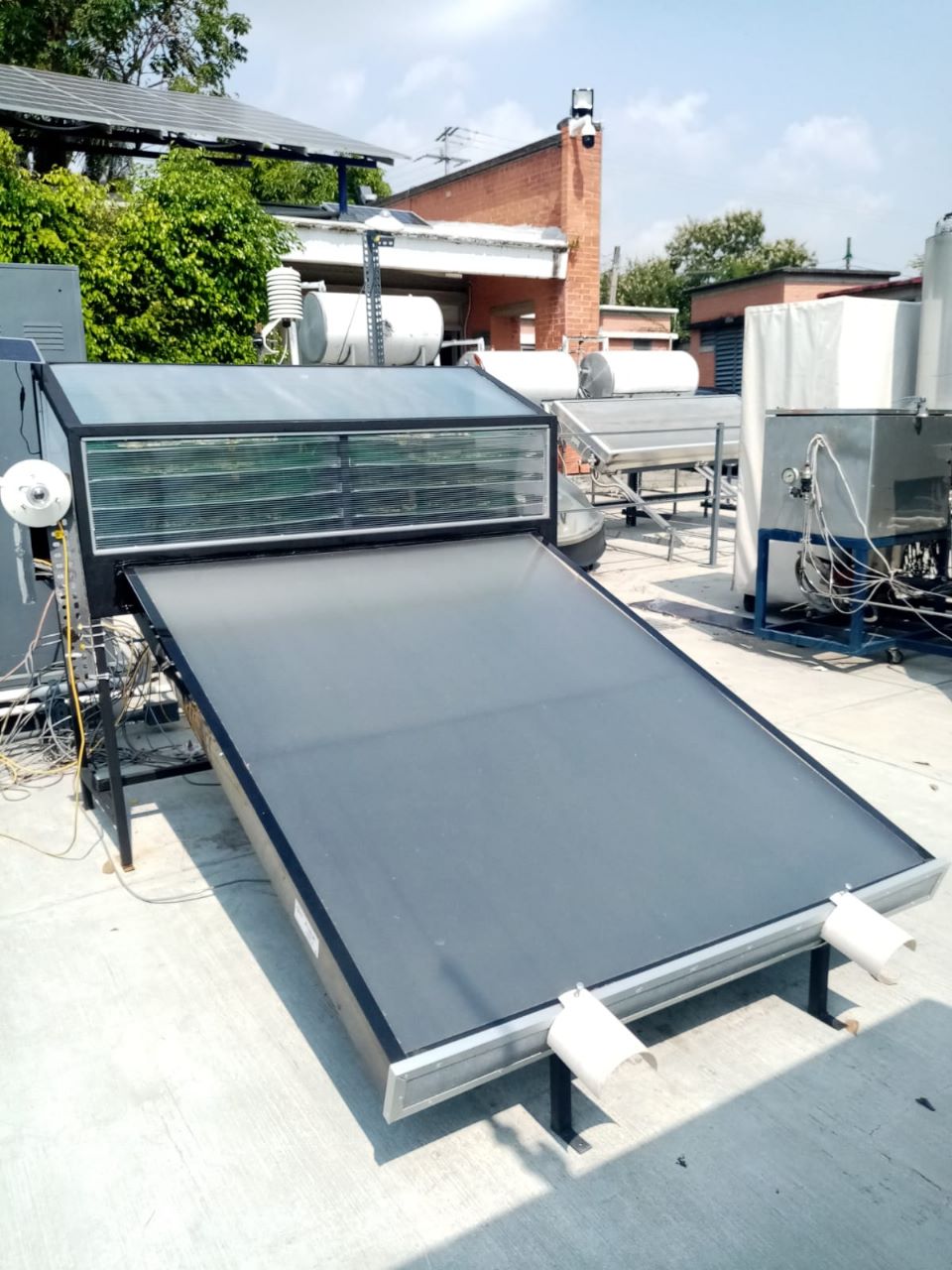

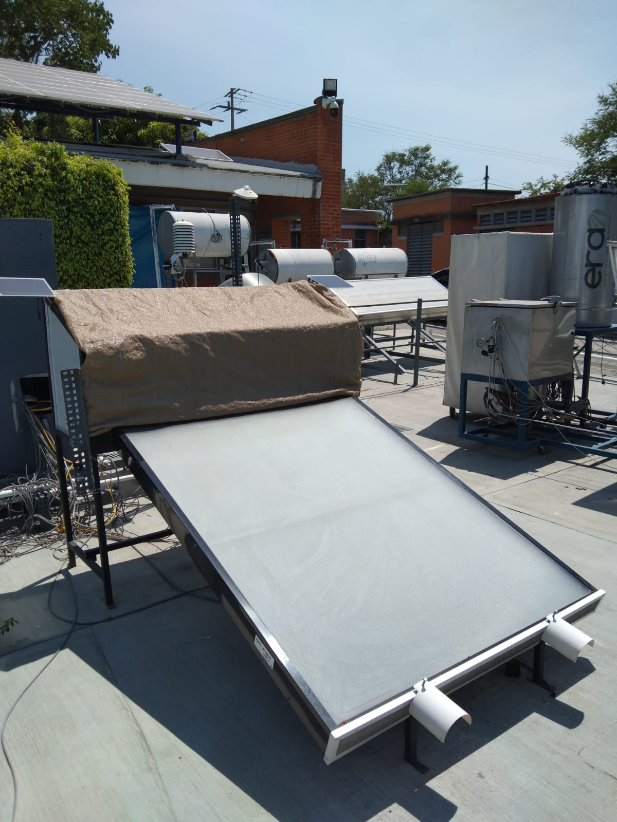


**A) B)**

**Fig. 1** Mixed-type solar dryer used in the drying process of Zompantle flower

***2.3 Instrumentation***

Two pyranometers (Kipp & Zonen, CM11;0-1400 W/m^2^ ± 2%, Netherlands) were placed to measure irradiance on drying days; one inclined in the plane of the collector and another vertical for the front area (south face) of the drying chamber. An ultraviolet pyranometer (Kipp & Zonen SUV5-V, Maximum UVA/UVB irradiance 400 W/m^2^, ± 2%, Netherlands) and 16 temperature sensors (RTD PT 1000, -50-750 °C ±0.2°C, Mexico) were placed, two at the inlet and two at the outlet of the collector, 10 in the trays inside the dryer and two at the outlet of the drying chamber. Measurements were recorded automatically using a data acquisition system (Agilent- 34972A) every 30 seconds. The air velocity was measured with two anemometers: a digital hot fin anemometer (Dwyer model 473B, ±0.1 m/s, USA) to measure fan air velocity and a hot wire anemometer (Dwyer model 471B, ±0.1 m/s, USA) for the air velocity at the outlet grilles; the tilt angle was 21°.

***2.4 Experimental design***

A 2^2^ factorial method was used. In this design, the operation mode (mesh shade and direct) and airflow (natural convection and forced convection) were established as factors (Supplementary Table 1). The levels of the elements were called "low" for mesh shade operation mode (MS) and natural convection (NC) and "high" for direct method (DM) and forced convection (FC). Four experimental tests were assessed, the experimental analysis was conducted in triplicate, and the data were analyzed using MINITAB 16 (Supplementary Table 2). The response variables were moisture content (Y_1_), water activity (Y_2_), lightness (Y_3_), chroma (Y_4_), hue (Y_5_), color difference (Y_6_), protein content (Y_7_), fat (Y_8_), fiber (Y_9_), ash content (Y_10_), and total soluble solids (Y_11_).

**Table 1** Experimental design 2^2^ in the solar drying of zompantle

| Factor | Level | |
| --- | --- | --- |
|  | (+) | (-) |
| Operation mode | Mesh shade | Direct |
| Airflow (m/s) | Forced convection | Natural convection |
|  | Experimental test |  |
| Experiment | Operation Mode | Airflow |
| 1 | - | - |
| 2 | + | + |
| 3 | - | + |
| 4 | + | + |

**Table 2** Analysis of variance of response variables

| Responses | | | | | |
| --- | --- | --- | --- | --- | --- |
| *Y_1_*=Moisture content | | | | | |
| Factor | DF | Sum of squares | Mean squares | F-Ratio | Prob. Level |
| X_1_ | 1 | 0.34340 | 0.34340 | 0.59 | 0.4657 |
| X_2_ | 1 | 8.28340 | 8.28340 | 14.15 | 0.0055* |
| X_1_X_2_ | 1 | 0.04208 | 0.04208 | 0.07 | 0.7955 |
| S | 8 | 4.68426 | 0.58553 |  |  |
| Total (adjusted) | 11 | 13.3530 |  |  |  |
| Total | 12 |  |  |  |  |
| *Y_2_*=Water activity | | | | | |
| X_1_ | 1 | 0.00294 | 0.00294 | 2.72 | 0.1376 |
| X_2_ | 1 | 0.00240 | 0.00240 | 2.22 | 0.1741 |
| X_1_X_2_ | 1 | 0.00681 | 0.00681 | 6.30 | 0.0364* |
| S | 8 | 0.00866 | 0.01082 |  |  |
| Total (adjusted) | 11 | 0.02083 |  |  |  |
| Total | 12 |  |  |  |  |
| *Y_3_*=Lightness | | | | | |
| X_1_ | 1 | 146.650 | 146.650 | 29.12 | 0.00064* |
| X_2_ | 1 | 14.2354 | 14.2354 | 2.83 | 0.13123 |
| X_1_X_2_ | 1 | 108.180 | 108.180 | 21.48 | 0.00167 |
| S | 8 | 40.2935 | 5.03669 |  |  |
| Total (adjusted) | 11 | 309.359 |  |  |  |
| Total | 12 |  |  |  |  |
| *Y_4_*=Chroma | | | | | |
| X_1_ | 1 | 524.966 | 524.966 | 323.68 | 0.00001* |
| X_2_ | 1 | 208.416 | 208.416 | 128.50 | 0.00003* |
| X_1_X_2_ | 1 | 628.576 | 628.576 | 387.56 | 0.00000* |
| S | 8 | 12.9751 | 1.62189 |  |  |
| Total (adjusted) | 11 | 1374.93 |  |  |  |
| Total | 12 |  |  |  |  |
| *Y_5_*=Hue | | | | | |
| X_1_ | 1 | 2.73607 | 2.73607 | 0.10 | 0.76417 |
| X_2_ | 1 | 466.378 | 466.378 | 16.42 | 0.00367* |
| X_1_X_2_ | 1 | 208.416 | 208.416 | 7.34 | 0.02669* |
| S | 8 | 227.158 | 28.3948 |  |  |
| Total (adjusted) | 11 | 904.689 |  |  |  |
| Total | 12 |  |  |  |  |
| *Y_6_*=*ΔE* | | | | | |
| X_1_ | 1 | 138.040 | 138.040 | 20.13 | 0.00203* |
| X_2_ | 1 | 64.8675 | 64.8675 | 9.46 | 0.01521* |
| X_1_X_2_ | 1 | 41.2923 | 41.2923 | 6.02 | 0.03968* |
| S | 8 | 6.85646 | 6.85646 |  |  |
| Total (adjusted) | 11 |  |  |  |  |
| Total | 12 |  |  |  |  |
| *Y_7_*=Proteins | | | | | |
| X_1_ | 1 | 0.46470 | 0.46470 | 38.82 | 0.00000* |
| X_2_ | 1 | 16.6323 | 16.1676 | 1350.0 | 0.00003* |
| X_1_X_2_ | 1 | 0.22060 | 0.22060 | 18.43 | 0.00000* |
| S | 8 | 0.02864 | 0.03589 |  |  |
| Total (adjusted) | 11 | 16.9487 |  |  |  |
| Total | 12 |  |  |  |  |
| *Y_8_*=Fat | | | | | |
| X_1_ | 1 | 0.60628 | 0.60628 | 31.47 | 0.00050* |
| X_2_ | 1 | 0.16317 | 0.16317 | 8.47 | 0.01958* |
| X_1_X_2_ | 1 | 1.27368 | 1.27368 | 66.11 | 0.00003* |
| S | 8 | 0.15411 | 0.01926 |  |  |
| Total (adjusted) | 11 | 2.19725 |  |  |  |
| Total | 12 |  |  |  |  |
| *Y_9_*=Fiber | | | | | |
| X_1_ | 1 | 1.09965 | 1.09965 | 74.59 | 0.000* |
| X_2_ | 1 | 0.00118 | 0.00118 | 0.080 | 0.784 |
| X_1_X_2_ | 1 | 0.36310 | 0.36310 | 24.63 | 0.001* |
| S | 8 | 0.11794 | 0.01474 |  |  |
| Total (adjusted) | 11 | 1.58188 |  |  |  |
| Total | 12 |  |  |  |  |
| *Y_10_*=Ash | | | | | |
| X_1_ | 1 | 0.49069 | 0.49069 | 55.66 | 0.00007* |
| X_2_ | 1 | 0.03658 | 0.03658 | 4.15 | 0.07601 |
| X_1_X_2_ | 1 | 0.14705 | 0.14705 | 16.68 | 0.00351* |
| S | 8 | 0.07053 | 0.00881 |  |  |
| Total (adjusted) | 11 | 0.74487 |  |  |  |
| Total | 12 |  |  |  |  |
| *Y_11_*=Total soluble solids | | | | | |
| X_1_ | 1 | 168.750 | 168.750 | 225.00 | 0.000* |
| X_2_ | 1 | 60.750 | 60.750 | 81.00 | 0.000* |
| S | 8 | 6.750 | 6.750 |  |  |
| Total (adjusted) | 11 | 236.250 |  |  |  |
| Total | 12 |  |  |  |  |
|  |  |  |  |  |  |

* Indicate significant differences at α=0.05

***2.4 Analytical methods***

The moisture content was obtained using a thermobalance (OHAUS, MB45, with a readability of 0.001 g) at 105 °C; around 3 g of the sample was placed and distributed uniformly on an aluminum pan inside the equipment. Water activity (aw) was determined with a Rotronic water activity meter (Higrolab C1) at 25 °C, where the disposable sample cup was covered entirely and introduced inside the kit for 20 minutes. The equipment was calibrated using Rotronic verification standards. Finally, the color properties were determined with a High-Quality Colorimeter (NR60CP+). The values were expressed as L (lightness), a (red-green), b (yellow-blue), H (hue angle), and C (chroma-saturation). From the results obtained, it was possible to calculate the color difference (∆E) between the raw and dried samples, chroma, and hue angle as reported by García [1]:

| $\Delta E = \left( {\Delta L}^{2}+ {\Delta a}^{2}+ {\Delta b}^{2} \right)^{\frac{1}{2}}$ |  |
| --- | --- |
| $C = \sqrt{{(a)}^{2} + {(b)}^{2}}$ |  |
| $H = arctg\left( \frac{b}{a} \right)$ |  |

The total soluble solids in raw and dried Zompantle flowers were determined by measuring the refractive juice index. The juice of the natural and dried Zompantle was prepared by adding 30 ml of distilled water to 10 g of Zompantle in an extractor machine; a drop of juice was placed into the prism of a hand refractometer and read as °Brix [1].

Protein content was determined by the standard Kjeldahl method, based on the quantification of total organic nitrogen by NMX-F-608-NORMEX-2011 [2]. 3-5 g of sample were used to digest organic material with concentrated sulfuric acid (analytical grade, JT Baker); then, the distillation was carried out using Kjeldahl equipment (GL-44, FERSA, Mexico). The distilled sample was titrated with 0.1 N hydrochloric acid (analytical grade, JT Baker). A factor of 6.25 was used to multiply by the percentage of nitrogen obtained and estimate the total crude protein.

Fat content was obtained by determining the Soxhlet gravimetric method using the ethereal extract. 1 to 2 g of samples were placed in a cartridge and introduced to the Soxhlet extractor, and 150 mL of ethyl ether (analytical grade, JT Baker) was added as an extraction solvent. Finally, the extraction was carried out for 6 hours, and the result was calculated using the NMX-F-615-NORMEX-2018 [3].

Total ash content was determined by complete calcination, weighing 3 to 5 g of sample into a porcelain crucible at constant weight and placing it on a grate to heat the sample slowly until no more smoke was released. The residue was removed to ashing in a muffle (RHF 1600, Carbolite, UK) at 550 °C, and the calculations were estimated according to NMX-F-607-NORMEX-2020 [4].

Crude fiber content was measured using the test method described in NMX-F-613-NORMEX-2017 [5]. First, 1 to 2 g of sample were subjected to acid digestion with hot sulfuric acid (analytical grade, JT Baker) at 0.255 N, the residue was filtered and washed with hot water, and later alkaline digestion was performed with sodium hydroxide (grade analytical, JT Baker) at 0.333 N. The residue was filtered and washed with hot water until neutral pH (7.0). Finally, the residue was dried at 100 °C for two hours and then calcined at 500 °C for one hour. All analytical determinations were carried out in triplicate.

Antioxidant activity was determined: Distilled water was added to 20 g of the sample, and the mixture was heated without boiling. Subsequently, samples were cooled down and filtered. After that, solids were frozen with liquid nitrogen, freeze-dried, and saved in vials for analysis. The antioxidant activity of the extracts was determined using the 2,2-diphenyl-1-picrylhydrazil (DPPH) free radical scavenging activity method, described by Chaves [6], with some modifications. 1.5 to 2 mg of samples were weighed and dissolved in deionized water to determine antioxidant activity to obtain a 20 mg/ml concentration. The assay was performed in 96 well plates. In these wells, 50 μl of the solution was placed at increasing concentrations from 1 to 100 μg/ml. Subsequently, 150 μl of 133 μM ethanolic DPPH solution (final concentration 100 μM) were added. The plate was incubated for 30 minutes at 37°C in the dark and under constant stirring. The absorbance of each well was measured at 515 nm in a microplate reader (Bio-Tek, Elx-808). The activity on DPPH is expressed as a percentage reduction and calculated with the following equation:

$$\% reduction=\left( \frac{C-E}{C} \right)*100$$

Where:

C = OD (Optical Density) of DPPH 100 μM

E = OD (Optical Density) of the DPPH mixture 100 μM + mixture

The total carbohydrates were determined based on the formula described by Fikiru [7]: Total carbohydrates (%) = 100 - (% Moisture + % protein + % crude fat + % natural fiber + % ash)

**Drying kinetic**

The solar drying process of Zompantle flower was carried out on the day: A) 31^st^ of January, B) 1^st^ – 2^nd^ of February, C) 7^th^-8^th^ of February, and D) 9^th^-10^th^ of February, 2023 by using an active dryer mixed type (Figure 2).

A)


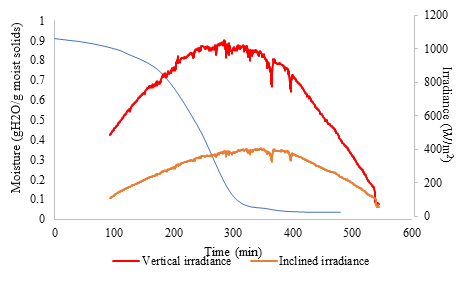


B)


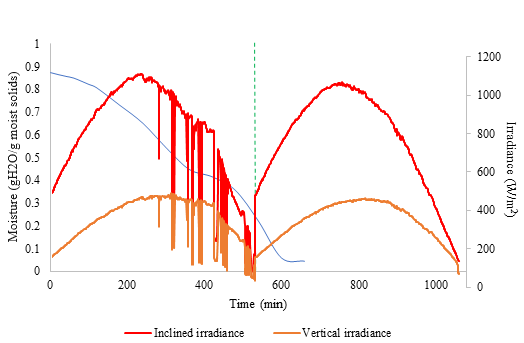


C)
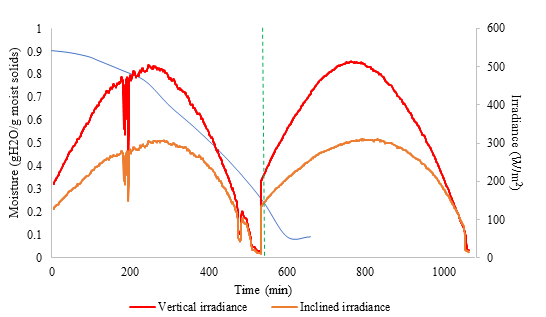


D)


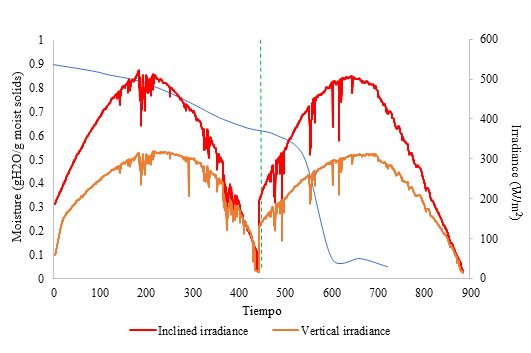


**Fig. 2** Drying kinetics of Zompantle (*Erythrina Americana)* carried out in a mixed-type solar dryer the day: A) 31^st^ of January 2023, B) 1^st^ – 2^nd^ of February, C) 7^th^-8^th^ of February, and D) 9^th^-10^th^ of February 2023

**Mathematical modeling**

On the other hand, the moisture content and moisture ratio during the process were calculated by applying the following equations:

| $M=\frac{w_{1-}w_{2}}{w_{2}}$ |
| --- |

| $MR=\frac{M-M_{e}}{M_{0}-M_{e}}$ |
| --- |

Where *M* is the moisture content, $w_{1}$ and $w_{2}$ are the initial and final sample weight, *MR* is the moisture ratio, $M_{e}$ and are the equilibrium moisture content and initial moisture. MR vs. time is used to compare the drying kinetics to the thin layer equations.

Supplementary Table 3 shows the thin layer drying equations for mathematical modeling tested to select the best model describing the drying curves from experimental results. The values of R^2^ were used to determine the fitting quality.

**Table 3** Theoretical models for adjusting experimental data

| **Model name** | **Equation** | **Reference** |
| --- | --- | --- |
| Weibull | $MR=exp({\frac{-t}{\beta})}^{\alpha}$ | Tzempelikos [8] |
| Modified Page | $MR=exp(-\left( kt \right)^{n})$ | White [9] |
| Page | $MR=exp({-kt}^{n})$ | Page [10] |
| Logarithmic | $MR=a\exp\left( -kt \right)+c$ | Togrul [11] |
| Wang & Sing | $MR=1+at+{bt}^{2}$ | [12] |

The coefficient values and the constants are presented in Supplementary Table 4. As can be seen, the maximum R^2^ is 0.9981, and the minimum is 0.9941; therefore, it can be concluded that all these models represent the behavior of the drying process and can be used to predict the solar drying of a pumpkin flower.

**Table 4** Modeling parameters for drying kinetics

| Operation Mode | Model adjusted | | | | | |
| --- | --- | --- | --- | --- | --- | --- |
|  | Weibull | Logarithmic | Wang & Sing | Modified Page | Page |  |
| MS-NC | R^2^ 0.9993  a -0.0079  b -1.0095  k 0.1497  n 1.4327 | --- | --- | R^2^ 0.9993  a ---  b ---  k 0.2689  n 1.4542 | R^2^ 0.9993  a ---  b ---  k 0.1481  n 1.4542 |  |
|  |  |  |  |  |  |  |
| DM-NC | R^2^ 0.9979  a 0.02360  b -0.9940  k 0.2067  n 1.6441 | --- | --- | R^2^ 0.9961  a ---  b ---  k 0.4063  n 1.6001 | R^2^ 0.9961  a ---  b ---  k 0.2366  n 1.6001 |  |
|  |  |  |  |  |  |  |
| MS-FC | R^2^ 0.9972  a -0.1229  b -1.1191  c ---  k 0.1662  n 1.0562 | R^2^ 0.9970  a 1.1690  b ---  c -0.1628  k 0.1725  n --- | R^2^0.9951  a -0.1707  b 0.0074  c ---  k ---  n --- | --- | --- |  |
|  |  |  |  |  |  |  |
| DM-FC | R^2^ 0.9982  a 0.00960  b -0.9820  k 0.20864  n 1.32345 | --- | --- | R^2^ 0.9981  a ---  b ---  k 0.30278  n 1.28053 | R^2^ 0.9981  a ---  b ---  k 0.2165  n 1.2805 |  |

MS= Mesh shade, NC= Natural convection, DM= Direct mode, FC= Forced convection

The dimensionless moisture ratio to observe the dehydration behavior of the Zompantle (MR) was calculated and fitted into different models (Figure 3).

A)


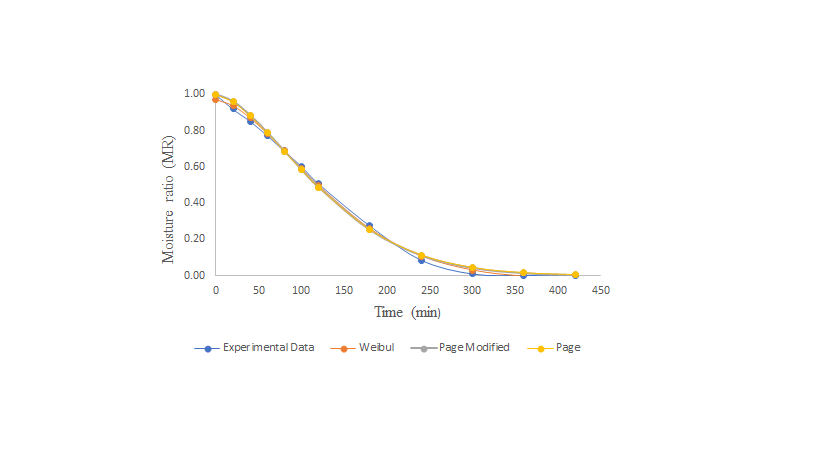


B)


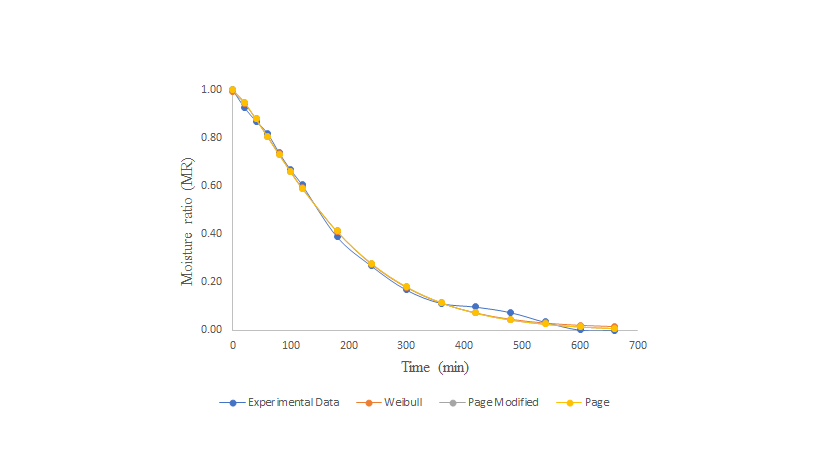


C)


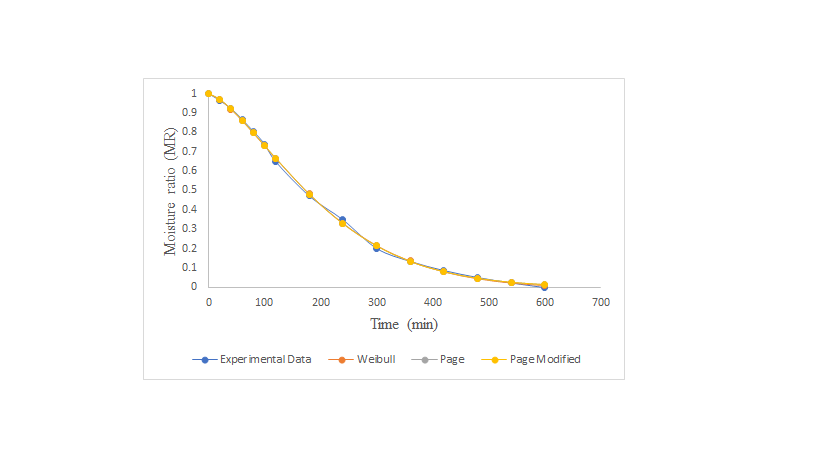


D)


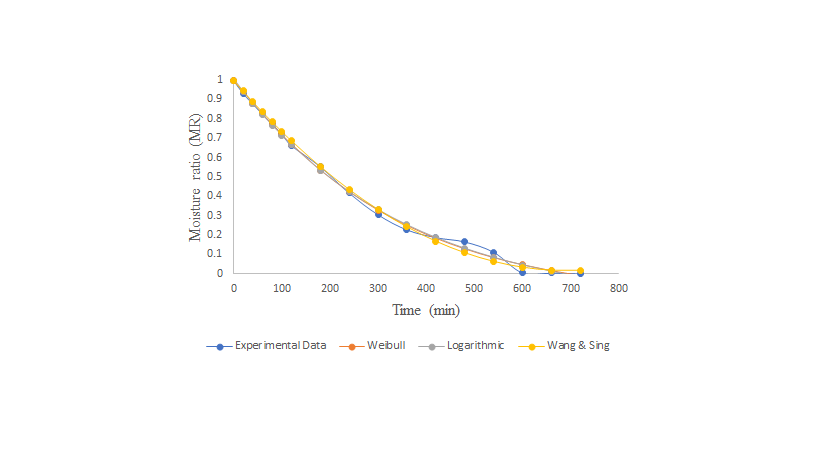


**Fig. 3** Mathematical fit of experimental drying kinetics of Zompantle (*Erythrina Americana)* carried out in a mixed-type solar dryer the day: A) 31^st^ of January 2023, B) 1^st^ – 2^nd^ of February, C) 7^th^-8^th^ of February, and D) 9^th^-10^th^ of February 2023

**Energy efficiency**

The drying experimental data obtained were used to perform the energy analyses of the Zompantle flower solar drying process.

**Thermal efficiency of the solar collector**

The energy efficiency in the collector was calculated by applying the following equation:

| $\eta_{c}=\frac{\dot{m}_{da}Cp_{da}\left( T_{out,C}-T_{in,C} \right)}{A_{C}I_{T}}$ |
| --- |

Where $\dot{m}_{da}$ is the air mass flow rate, $Cp_{da}$ is the specific heat of air at constant pressure at an average temperature between the inlet and outlet of the solar collector, $T_{out,C}$ and $T_{in,C}$ is the temperature at the outlet and inlet of the collector, respectively. While $I_{T}$ is the incident solar radiation on the plane of the collector and $A_{C}$ is the gross solar collector area.

**Thermal efficiency of the dryer**

In a solar dryer, energy efficiency relates to the energy used for the evaporation of moisture contained in food at a given temperature with the total power supplied to the dryer [13]. The energy efficiency of the dryer was calculated by applying the following equation:

| $\eta_{E}=\frac{m_{w}h_{fg}}{\left( A_{C}*I_{PI} \right)+A_{G_{T}}\left( I_{PI}*F_{EI} \right)+{A_{G}}_{V}{(I}_{PV}*F_{EI})+W_{vent}}$ |
| --- |

Where $m_{w}$ is the mass of water evaporated, $h_{fg}$ is the latent heat of vaporization of water, $A_{C}$ is the gross solar collector area, ${A_{G}}_{T}$ is the inclined area of the drying chamber corresponding to the roof, $I_{PI}$ is the irradiance in the plane of the collector. ${A_{G}}_{V}$ is the vertical area of the chamber corresponding to the front multiplied by $I_{PV}$ which is the irradiance in the vertical plane, $W_{vent}$ is the fan power and $F_{EI}$ is the fraction of incident energy.

**Total thermal efficiency of the drying**

The drying efficiency relates to the energy used to heat the food and evaporate the water present in it, with the energy supplied to the drying device [14]. It is calculated with the following equation:

| $\eta_{D}=\frac{m_{p}Cp_{p}\left( T_{p,t+dt}-T_{p,t} \right)+m_{w}h_{fg}}{\left( A_{C}\eta_{C}*I_{PI} \right)+\left( I_{PI}*F_{EI} \right)\left( A_{G_{T}}\tau_{G} \right)+{A_{G}}_{V}{(I}_{PV}*F_{EI})\tau_{G}}$ |
| --- |

Where $m_{p}$ and $Cp_{p}$ are mass and specific heat of the food, respectively. $T_{p,t+dt}$ is the temperature of the product in the next time step, $T_{p,t}$ is the temperature at that moment, $\eta_{C}$ is the collector efficiency and $\tau_{G}$ is the transmittance of the drying chamber cover.

The results of the collector, dryer, and drying efficiencies for the four experiments are shown in Supplementary Table 5.

The results showed the highest collector efficiency (21.83 and 31.05%) when the dryer was operated with forced convection. On the other hand, the efficiency was 4.25 and 8.60% with natural convection. The dryer efficiency ranged from 3.50 to 5.72% with MS-NC (Mesh shade-natural convection) and MS-FC (Mesh shade-forced convection). Finally, the total drying efficiency was 14.84% with the direct mode and natural convection (DM-NC) and 17.10% with the mesh shade and natural convection (MS-NC). As seen from the Table, the efficiency increases when the mesh shade is used.

According to López Vidaña [14], the high values of drying efficiency when a cover was used in the chamber, are because the energy that reached the dryer was used in a more significant proportion than when there was no cover, where the efficiencies were lower since there is more energy available because solar radiation enters directly into the drying chamber.

**Table 5** Results obtained for the efficiencies.

| Operation mode | Efficiency (%) | | |
| --- | --- | --- | --- |
|  | Collector | Dryer | Drying |
| MS-NC | 8.60 | 3.50 | 17.10 |
| DM-NC | 4.25 | 3.95 | 14.84 |
| MS-FC | 31.05 | 5.72 | 7.30 |
| DM-FC | 21.83 | 3.61 | 5.93 |

MS= Mesh shade, NC= Natural convection, DM= Direct mode, FC= Forced convection

References

1. García O, Lucho A, Montiel E, Castañeda M, Ortiz C, Castillo B, Luna G, Domínguez A (2023) Effect of modified solar dryers on colorimetric and physicochemical properties of pumpkin flower (*Cucurbita maxima*). Plant Foods Hum Nutr 78:139-145. <https://doi.org/10.1007/s11130-022-01032-8>
2. NMX-F-608-NORMEX-2011 Alimentos-determinación de proteínas en alimentos-método de ensayo. DOF - Diario Oficial de la Federación. (2014). Dof.gob.mx. <https://www.dof.gob.mx/nota_detalle.php?codigo=5360486&fecha=18/09/2014#gsc.tab=0>
3. NMX-F-615-NORMEX-2018 Alimentos-determinación de extracto etéreo (Método SOXHLET) en alimentos. DOF - Diario Oficial de la Federación. (2019). Dof.gob.mx. <https://dof.gob.mx/nota_detalle.php?codigo=5552291&fecha=08/03/2019#gsc.tab=0>
4. NMX-F-607-NORMEX-2020 Alimentos-determinación de cenizas en alimentos. DOF - Diario Oficial de la Federación. (2022). Dof.gob.mx. <https://dof.gob.mx/nota_detalle.php?codigo=5641716&fecha=01/02/2022#gsc.tab=0>
5. NMX-F-613-NORMEX-2017 Alimentos-determinación de fibra cruda en alimentos. DOF - Diario Oficial de la Federación. (2017). Dof.gob.mx. <https://www.dof.gob.mx/nota_detalle_popup.php?codigo=5534254>
6. Chaves, N.; Santiago, A.; Alías, J.C. Quantification of the antioxidant activity of plant extracts: analysis of sensitivity and hierarchization based on the method used. *Antioxidants.* **2020**, 9. DOI: 10.3390/antiox9010076
7. Fikiru O, Bultosa G, Fikreyesus S, Temesgen M (2016) Nutritional quality and sensory acceptability of complementary food blended from maize (Zea mays), roasted pea (Pisum sativum), and malted barley (Hordium vulgare). Food Science & Nutrition 5:173-181.

<https://doi.org/10.1002/fsn3.376>

1. Tzempelikos D, Vouros A, Bardakas A, Filios A, Margaris D (2015) Experimental study on convective drying of quince slices and evaluation of thin-layer drying models. Eng Agric Environ Food 8:169–177. <https://doi.org/10.1016/j.eaef.2014.12.002>
2. White G, Ross I, Poneleit C (1981) Fully-exposed drying of popcorn. Paper-American Society of Agricultural Engineers 24:466-469. <https://doi.org/10.13031/2013.34276>
3. Page G (1949) Factors influencing the maximum rates of air drying shelled corn in thin layers. Thesis Purdue University, West Lafayette, IN, USA
4. Togrul H (2005) Simple modeling of infrared drying of fresh apple slices. J Food Eng 71:311-323. <https://doi.org/10.1016/j.jfoodeng.2005.03.031>
5. Wang C, SinghR (1978) A single layer drying equation for rough rice. Am Soc Agric Eng 3001. https://doi.org/10.1081/E-EEE2-120046011
6. López E, César A, O. García O, Pilatowsky I, Brito R (2020) Thermal performance of a passive, mixed-type solar dryer for tomato slices (*Solanum lycopersicum*). Renew Energ 147: 845-855. https://doi.org/10.1016/j.renene.2019.09.018
7. López E, César A, García O, Salgado O, Domínguez A (2021) Energy and exergy analyses of a mixed-mode solar dryer of pear slices (*Pyrus communis* L). Energy 220. <https://doi.org/10.1016/j.energy.2020.119740>
